# Muscle synergies for multidirectional isometric force generation during maintenance of upright standing posture

**DOI:** 10.1007/s00221-024-06866-z

**Published:** 2024-06-14

**Authors:** Andrea Monte, Anna Benamati, Agnese Pavan, Andrea d’Avella, Matteo Bertucco

**Affiliations:** 1 Department of Neurosciences, Biomedicine and Movement Sciences, University of Verona, Via Felice Casorati 43, 37131 Verona, Italy; 2 Laboratory of Neuromotor Physiology, IRCCS Fondazione Santa Lucia, Rome, Italy; 3 Department of Biology, University of Rome Tor Vergata, Rome, Italy

**Keywords:** Muscle synergies, Postural control, EMG, Non-negative matrix factorization, Upright standing

## Abstract

Muscle synergies are defined as coordinated recruitment of groups of muscles with specific activation balances and time profiles aimed at generating task-specific motor commands. While muscle synergies in postural control have been investigated primarily in reactive balance conditions, the neuromechanical contribution of muscle synergies during voluntary control of upright standing is still unclear. In this study, muscle synergies were investigated during the generation of isometric force at the trunk during the maintenance of standing posture. Participants were asked to maintain the steady-state upright standing posture while pulling forces of different magnitudes were applied at the level at the waist in eight horizontal directions. Muscle synergies were extracted by nonnegative matrix factorization from sixteen lower limb and trunk muscles. An average of 5-6 muscle synergies were sufficient to account for a wide variety of EMG waveforms associated with changes in the magnitude and direction of pulling forces. A cluster analysis partitioned the muscle synergies of the participants into a large group of clusters according to their similarity, indicating the use of a subjective combination of muscles to generate a multidirectional force vector in standing. Furthermore, we found a participant-specific distribution in the values of cosine directional tuning parameters of synergy amplitude coefficients, suggesting the existence of individual neuromechanical strategies to stabilize the whole-body posture. Our findings provide a starting point for the development of novel diagnostic tools to assess muscle coordination in postural control and lay the foundation for potential applications of muscle synergies in rehabilitation.

**Supplementary Information:**

The online version contains supplementary material available at 10.1007/s00221-024-06866-z.

## Introduction

Upright standing is one of the most common postures by which humans interact with the environment. From a biomechanical viewpoint, the bipedal quiet standing posture can be described by an inverted pendulum (Winter et al. 1998; Peterka 2002, 2018), where the torque generated by lower-limb joints (i.e., ankle, knee and hip) is directly involved in keeping the projection of their center of mass within the base of support (Aramaki et al. 2001; Creath et al. 2005; Hsu et al. 2007; Pinter et al. 2008; Kuo 1995; Bloem et al. 2000; Krishnamoorthy et al. 2003; Alexandrov et al. 2005).

In this regard, previous studies have shown that postural muscles, that have different action directions, are co-activated in multi-directional and intentional movement of the CoP during upright standing (Kutch et al. 2008; Hagio and Kouzaki 2015). Specifically, the authors found that individuals had activation directions of lower limb muscles in either antero-posterior, medio-lateral or diagonal directions, such that CoP displacement was accomplished by the collaboration of multiple muscles. Therefore, humans coordinate the activation of several postural muscles that contribute to the multi-joint postures of lower limbs segments to accomplish the multidirectional control of upright standing (Grüneberg et al. 2005; Bingham et al. 2011; Forghani et al. 2017; Yamagata et al. 2019, 2021).

The control of standing posture involves multiple muscles around several joints; therefore, the central nervous system (CNS) must coordinate the many degrees of freedom of the musculoskeletal system, taking into consideration the nonlinear characteristics of the muscles and tendons and the dynamic interactions among the articulated segments of the body. To cope with this challenge, over the last two decades it has been proposed that the CNS relies on a modular organization in which movement is achieved by the combination of multiple muscle synergies (Bizzi et al. 1991; d’Avella et al. 2003; d’Avella and Bizzi 2005). Indeed, muscle synergies have been proposed as building blocks employed by the CNS to simplify the generation of task-specific forces and movements with a redundant neuromuscular system, by means of coordinated recruitment of groups of muscles with specific activation balances or temporal profiles (Bizzi et al. 2002; d’Avella and Bizzi 2005; d’Avella et al. 2011). By combining an adequate number of motor subtasks, the CNS achieves movement translating high-level and low-dimensional neural commands into low-level and high-dimensional patterns of muscle activity. On this matter, muscle synergies have been reported in several motor behaviors and tasks, including running (Nishida et al. 2017; Saito et al. 2018; Bach et al. 2021), walking (Ivanenko et al. 2004; Cappellini et al. 2006; Chvatal and Ting 2012, 2013), kicking (d’Avella et al. 2003), writing (Lunardini et al. 2015, 2017) and reaching tasks (d’Avella et al. 2006, 2008, 2011). Muscle synergies in postural control have also been examined in numerous studies (Krishnamoorthy et al. 2003; Torres-Oviedo et al. 2006; Torres-Oviedo and Ting 2007, 2010; Klous et al. 2010; Chvatal and Ting 2013; Safavynia and Ting 2013; Piscitelli et al. 2017).

As far as it is concerned postural control, comprehensive investigation of neuromechanical characteristics pertaining to muscle synergies during the state of quiet upright standing posture remains relatively unexplored. Previous studies investigated muscle synergies primarily in automatic postural responses to external or self-initiated perturbations (see for example: Krishnamoorthy et al. 2003; Torres-Oviedo et al. 2006; Torres-Oviedo and Ting 2007; Klous et al. 2010; Chvatal and Ting 2013; Safavynia and Ting 2013; Piscitelli et al. 2017).

Only a recent study examined the action directions of postural synergies in the voluntary multidirectional control of standing posture (Kubo et al. 2017). The results indicated that muscles synergies were distributed in well-balanced directions, suggesting that they contribute to the simplification of postural control by reducing the redundancy caused by a large number of muscles involved in bipedal standing. However, participants were instructed to move their CoP from the steady-state upright initial position to one of the target points, evenly spaced in the horizontal plane, by leaning their body around the ankle joint and holding the position at the target with a CoP displacement of 3 cm. In other words, the participants generated a volitional muscle torque around the ankle joint to transfer their body to a different configuration of spatial reference coordinates. This postural task is different from typical steady-state upright standing behaviors, in which individuals are required to continuously activate muscles to generate appropriate joint torques that correct for the deviation of CoP fluctuations due to the inherent instability of quiet standing posture and, thus, to maintain the projection of their center of mass on the ground within the base of support (Peterka 2002, 2018). Thus, the neuromechanical characterization of muscle synergies during quiet upright standing posture is still incomplete. Such investigation would provide enhanced insights into the neural organization of the central nervous system governing postural control. Additionally, it would ascertain whether the activation of postural muscles can be effectively represented by a limited set of muscle synergies specifically attuned to the multidirectional control inherent in maintaining an upright standing posture.

Previous studies have demonstrated that tasks involving the generation isometric multidirectional force can be used for extracting synergies (Borzelli et al. 2013; Berger et al. 2013; Gentner et al. 2013; Barradas et al. 2020; Borish et al. 2021; Cho et al. 2022). Therefore, the aim of the study was to investigate muscle synergies while maintaining and controlling the CoP fluctuations during upright standing posture in which participants were instructed to generate isometric forces to counteract small loads pulling in multiple directions. We hypothesized that the muscle activation patterns during upright standing posture will be reproduced by the independent modulation of a small set of muscle synergies with a different amplitude related to the pulling force directions and loads.

## Materials and methods

### Participants

Eleven healthy adults participated to the study (7 males and 4 females, 24.2 ± 0.1years old, body mass 70 ± 12 kg, height 172 ± 6 cm). None of the participants had previous history of neurological or musculoskeletal injuries. All participants provided written informed consent before taking part in the experimental procedures. The study protocol was performed in accordance with the Declaration of Helsinki, and was approved by the Ethics Approval Committee for Human Research of the University of Verona (Approval number 22/2020).

### Experimental protocol

In this study we developed a new-experimental set-up to investigate the structure and recruitment of muscle synergies used to maintain upright balance during external perturbing pulling forces.

**Fig. 1:**
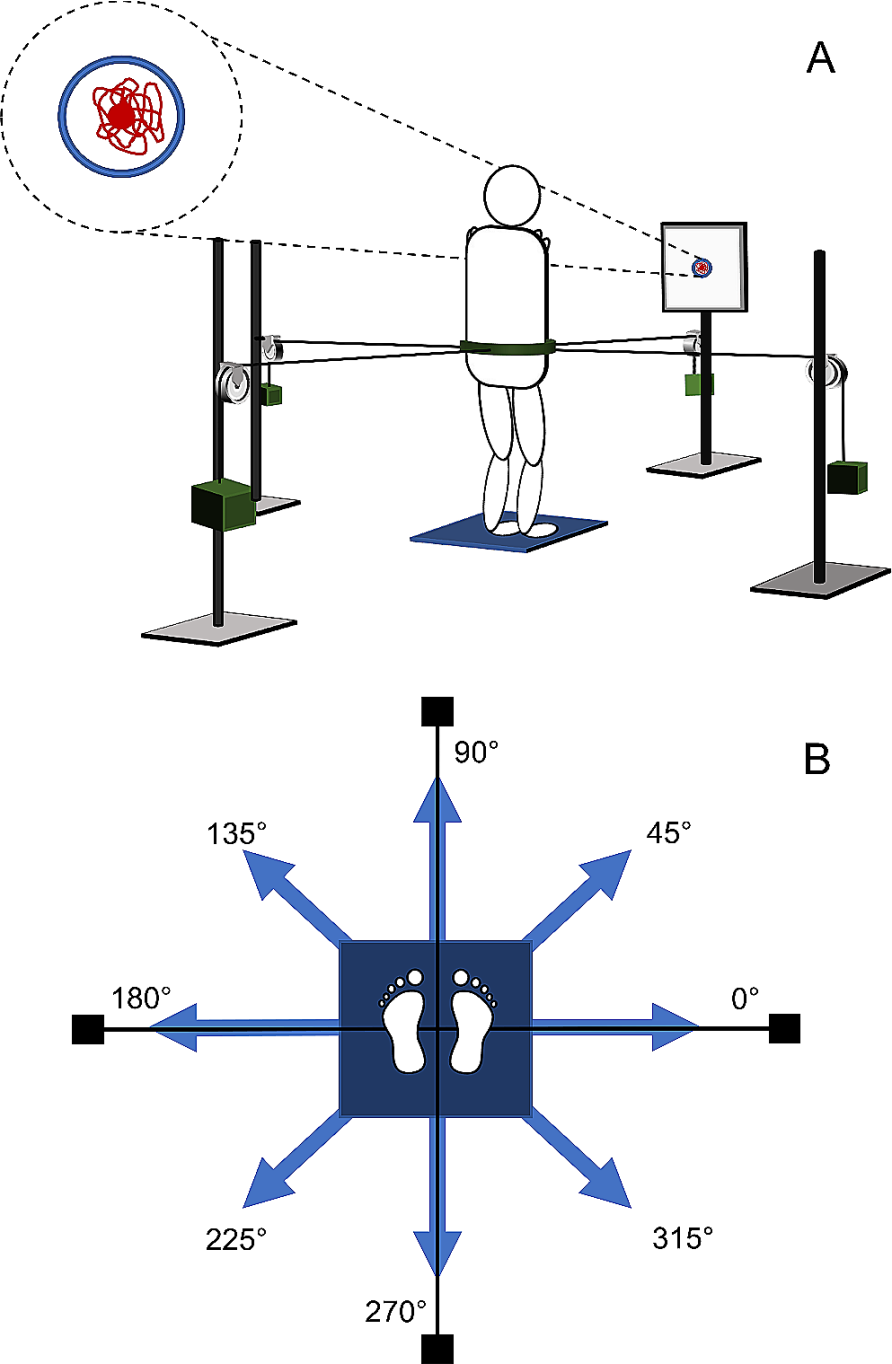
**(A)** experimental set-up rear view; **(B)** pulling forces directions. The participant stood on the platform with the hands positioned at the level of the shoulders while looking at the monitor where real-time feedback (represented by the dot) and the target (represented by the circle) were provided. After 5 s of standing position a pulling force (one of those reported in panel B) was applied

One single experimental session was conducted for each participant. They were asked to maintain their normal standing position on a force platform while looking at a monitor where real-time feedback of the 2-D position of their CoP was provided. The feedback was a red circular cursor of 0.5 cm diameter displayed on the monitor positioned 1.5 m in front of the subject (see Fig. 1-A) and with a refresh rate of 12 Hz. Participants positioned their hands at the level of the shoulders to avoid any contribution/assistance of the arms’ motion.

After 5 s of standing position, a pulling force of one of two different magnitudes (5% or 10% of body weight; BW) was applied at the level of the waist (Fig. 1-A) by means of a cable. To do that, an inelastic belt with four hooks was worn at the hip level. The hooks were attached to the belt, as reported in Fig. 1-A, and four different poles positioned in front, behind, to the left and right side of the subject with a pulley and a weight support were utilized for applying the pulling force. These specific loaded conditions were selected after several pilot experiments, where different loaded conditions were tested. These loads, expressed as percentages of the body weight, are indeed able to elicit significant variations of the EMG amplitude in the recruited muscles, avoiding angular changes in the lower limb joints.

As the pulling force was applied, participants were instructed to maintain the starting position by keeping the red circular cursor within a blue circular target 12.5 mm in radius without loss of balance. Loss of balance was defined as stepping with at least one foot during the application of the external force. The trial ended when cursor had remained in the target for at least 10 consecutive seconds (Fig. 1-A). Typical trial duration was 20 s (5 s of baseline within the target, 5 s to reach the target after perturbation, 10 s within the target while maintaining a static posture counteracting the load. After that, the load was removed. To counteract the load, participants were required to contract the muscles. However, they were not allowed to move their feet. As their posture did not change, an isometric force was generated by leg and trunk muscles.

The pulling force was applied in eight evenly spaced directions in the horizontal plane: 0°, 45°, 90°, 135°, 180°, 225°, 270° and 315° (where 0° corresponds to the right side of the participant; see Fig. 1-B for further details). Each direction was repeated 5 times with two load conditions: 5% BW (Light) and 10% BW (Heavy) of participant’s body weight (BW). A total of 80 trials were conducted from each subject in a randomized order. Randomization was conducted using a specific online software (Randomizer.com). The randomization was conducted across series (5 series x 2 load x 8 directions). Passive recovery was adopted to avoid fatigue between trials.

### Apparatus

During each trial the CoP displacement and the Ground Reaction Forces (GRF) in the anteroposterior (CoPx, Fx) and mediolateral (CoPy, Fy) directions were recorded using a force platform (AMTI, USA, 90 × 90 cm, sampling frequency at 1000 Hz).

The EMG activity of 16 muscles was recorded (ZeroWire, Aurion, Italy, sampling at 1000 Hz) from the right side of each participant. The muscles included: rectus abdominis (RA), tensor fasciae latae (TFL), biceps femoris long head (BF), tibialis anterior (TA), semitendinosus (SMT), semimembranosus (SMB), rectus femoris (RF), peroneus (PER), medial gastrocnemius (GM), lateral gastrocnemius (GL), erector spinae (ES), external oblique (EOB), gluteus medius (GLT), vastus lateralis (VL), vastus medialis (VM), and soleus (SOL). In this experimental set-up we investigated only one side of the body, assuming a symmetric activation of the other sides in accordance with previous studies (Torres-Oviedo and Ting 2007, 2010; Kubo et al. 2017).

All instruments (EMG, CoP, GRF, and real-time biofeedback) were synchronized. For each trial a data matrix with 16 EMG channels, GRF, and CoP displacement as a function of time was obtained.

### Data processing

All data processing was performed using Matlab software (version 9.12, MathWorks, Natick, MA, USA), RStudio software (RStudio Inc., Version 1.4.1103, Boston, MA, USA) and SPSS software (version 16.0, IBM Corp., Armonk, NY, USA).

Each trial showed a *loading phase* (where the load was applied and the subject counteracted it), a *steady-state phase* (where the subject remained with the CoP inside the target for at least 10 s) and an *unloading phase* (where the load was removed). Since we were not interested in the transient phases (*loading* and *unloading phases*), the raw data were analyzed only in the *steady-state phase* by means of custom Matlab routines. The GRF and CoP data were filtered with a zero-phase-lag 4th order Butterworth low-pass filter with a cut-off frequency of 30 Hz to remove the effects of postural adjustment and signal fluctuations that are not related to physiological parameters (e.g., as in the case of behavioral noise). For each trial, the two-dimensional GRF (i.e. combined Fx and Fy components) was calculated and normalized by the participant’s BW to check the real force applied during the steady-state phase (*nGRF*). The mean value of the resultant force was calculated during the steady-state phase as an indicator of the trial quality.

The CoP data was analyzed frame-by-frame using a Euclidean distance approach after filtering. We calculated the point-by-point distance from the center of the target to the CoP coordinates (i.e., the center of the red cursor) as an indicator of performance. After that, the mean values of the Euclidean distances obtained during the steady-state phase were calculated. In this regard, we calculated the *Error* performed by the subject during the task as the ratio between the Euclidean distance (in millimeters) and the radius dimension (12.5 mm). Therefore, values larger and lower than 1 indicated that the participant was outside or inside the target, respectively. Trials were considered successful when the error was lower than 1 (one) (i.e., indicate that the participant remained within the target). The effects of loading conditions and direction of pulling forces on *nGRF* and *Error* were tested with a two-way repeated-measures ANOVA with fixed effect as *Load* (2 levels: 10% BW and 5% BW) and *Direction* (8 levels: 0º to 315º). Whenever the Mauchly’s test of sphericity was not satisfied, the Greenhouse–Geisser correction for the degrees of freedom was applied. Pairwise comparisons with Tukey corrections were used to explore significant effects.

Raw EMG data were high-pass filtered at 35 Hz, de-meaned, rectified, and low-pass filtered at 40 Hz (Ting and Macpherson 2005). The EMG data were further integrated over 10-ms intervals to reduce the size of the data set. Only data obtained in the *steady-state phase* were utilized for further analysis. The EMG values of each muscle during the *steady-state phase* was normalized to the maximum value across all loads and directions (Torres-Oviedo and Ting 2007, 2010; Berger and d’Avella 2014). Finally, the mean value was calculated.

The variations of the muscle patterns across the experimental conditions were analyzed by identifying muscle synergies from the mean EMG activity collected during the steady-state phase. For each participant we obtained a set of 80 vectors, each representing the average activity of all recorded muscles for each trial in one experimental condition (8 directions × 2 loads × 5 trials). We then used a nonnegative matrix factorization (NMF) algorithm (Lee and Seung 1999; Ting and Macpherson 2005; d’Avella and Bizzi 2005; Thresh et al. 2006) to decompose each one of these muscle activity vectors (**m**) as the combination of a unique set of N time-invariant synergies (***w***_*i*_) multiplied by condition-specific scaling activation coefficients (*c*_*i*_):

1 $$\varvec{m}= \sum _{i=1}^{N}{c}_{i} {\varvec{w}}_{i}+{\varvec{e}}_{m}$$

where ***e***_*m*_ is an N-dimensional vector of muscle activation residuals. Different matrix factorization algorithms have been used for the identification of muscle synergies, assuming that the observed EMG data can be modeled as a linear combination of a small set of basis vectors (Tresch et al. 2006). To assess the potential bias towards the heavy load condition (10% BW), muscle synergies were additionally extracted by normalizing the EMG values to the maximum value of 5% and 10% BW separately.

The NMF decomposition technique assumes that each muscle activation pattern is decomposed in a linear combination of a few non-negative muscle synergies and non-negative synergy activation coefficients. A small set of muscle synergies states that representing muscle activity vectors (**m**) in terms of the vectors ***w***_*i*_ and scaling activation coefficients *c*_*i*_ is lower-dimensional because it requires a lower number of values than the number of values of all elements of the **m** vectors. In particular, such linear decomposition technique states that over a large number of observations **m** the components ***w***_*i*_ remain fixed, but the scaling activation coefficients *c*_*i*_ are allowed to change and are sufficient to account for all variations in the data measured across different motor task conditions.

To extract a set of synergies, the iterative decomposition algorithm was initialized with random values for synergies and coefficients, and it stopped when the reconstruction R^2^ value increased by < 10^− 4^. At each iteration the algorithm performed two steps: (1) it updated the synergies given the data and the coefficients; (2) it updated the coefficients given the synergies and the data. The extraction was repeated 40 times with random initial conditions and only the synergy set with the highest R^2^ was retained. Then, final number of synergies was selected as in d’Avella et al. (2006), according to the curve of the reconstruction R^2^ as a function of N (i.e., number of set of synergies extracted). The number of synergies at which the curve of R^2^ showed a change in slope, indicating that adding additional synergies did not significantly improve the accuracy of the reconstruction (Tresch et al. 2006; d’Avella et al. 2006; Ranaldi et al. 2021), was selected as the optimal number of synergies. In this regard, assuming that the R^2^ follows a straight line, it is possible to identify the value of N after which the R^2^ curve is essentially straight. Therefore, a series of linear regressions, firstly taking into account the entire R^2^ curve and progressively removing one synergy from the regression interval. The mean square residual errors of the different regressions was calculated and used a determinant for the synergy number. Indeed, the optimal number of synergies was obtained when the mean square error was lower than 10^− 3^.

We examined whether the muscle synergies extracted by our algorithm were influenced by any inherent bias built into the method by comparting the R^2^ value for the reconstruction of the real data with the extracted synergies and the R^2^ value for the reconstruction of structureless simulated data with synergies extracted from those simulated data. The generation of structureless data involved a process whereby the samples for each muscle were randomly reshuffled independently of the muscle activation data matrix (**m**). Consequently, the resultant simulated data exhibited the same muscle amplitude distribution as the real data, but each muscle amplitude was uncorrelated with all the others. This verification was achieved by comparing the R^2^ values obtained from reconstructing real data with the extracted synergies against those derived from reconstructing structureless simulated data. For each simulated dataset we repeated 100 synergy extraction runs with the same procedure used for the real data (d’Avella and Bizzi 2005; Borzelli et al. 2013).

The effects of loading conditions and direction of pulling forces on the activation coefficients were tested with a two-way repeated-measures ANOVA with fixed effect as *Load* (2 levels: 10% BW and 5% BW) and *Direction* (8 levels: 0º to 315º). The Greenhouse–Geisser correction for the degrees of freedom was applied in case the Mauchly’s test of sphericity was not satisfied. Pairwise comparisons with Tukey corrections were used to explore significant effects.

We characterized the directional tuning of the synergy amplitude coefficients with a cosine function (d’Avella et al. 2006, 2008). Briefly, for each participant, synergy and load; we performed a multiple linear regression (Matlab function “regress”) to fit the following model.

2 $$c\left(\theta \right) = {\beta }_{0} + {\beta }_{x}cos\theta + {\beta }_{x}sin\theta$$

where *q* is the direction of the perturbation, *c* is the synergy amplitude coefficient, and *β*_*0*_ is an *Offset* parameter. We then computed the *Amplitude, r*:

3 $$r= \sqrt{{\beta }_{x}^{2}+ {\beta }_{y}^{2}}$$

and the preferred direction, *θ*^*PD*^:

4 $${\theta }^{PD}= {\text{tan}}^{-1}\left({\beta }_{y} / {\beta }_{x}\right)$$

and re-wrote Eq. 2 as a cosine tuning function:

5 $$c\left(\theta \right)= {\beta }_{0}+r \text{cos}\left(\theta - {\theta }^{PD}\right)$$

The goodness of the fit was quantified with the r^2^ value of the multiple linear regression, and its significance was tested with an *F* test. We also quantified the variability of the preferred direction (θ^PD^) across conditions by computing its angular deviation (*AngDev*) (d’Avella et al. 2008), defined as the square root of 2(1 − q), where q is the length of the vector resulting from the sum of unit vectors directed as the preferred directions divided by the number of vectors (*angular.deviation* function, R-package ‘circular’).

To compare the synergies extracted from different participants, we grouped them using hierarchical clustering. We used the similarity between a pair of synergies (*S*_*ij*_), computed with the subset of muscle common to all participants, to define a distance measure (dij = 1 − Sij) (Matlab function “*pdist*”) with the “cosine” method. Then, we created a hierarchical cluster tree from all synergy pairs (Matlab function “*linkage*” with the “average” distance method, i.e. using as distance between two clusters the average distance between all pairs of objects across the two clusters). The cophenetic correlation coefficient was calculated to measure how accurately the tree represented the dissimilarities between observations (Matlab function “*cophenet*”). We partitioned the hierarchical tree with the minimum number of clusters for which there was no more than one synergy from the same participant in each cluster (Matlab function “*cluster*”) (d’Avella et al. 2006). As an alternative approach, we determined the number of clusters by identifying the largest vertical difference between nodes in the dendrogram from the hierarchical clustering and drew a horizontal line across the midpoint. Then, the optimal number of clusters was determined by counting the number of vertical lines intersecting with the horizontal line. Hereafter, we defined the clustered synergies as *cW* to differentiate them from the *w* that resulted from the NMF for each participant; accordingly, the corresponding grouped scaling activation coefficients were defined as *cC*.

## Results

### Task performance

**Fig. 2:**
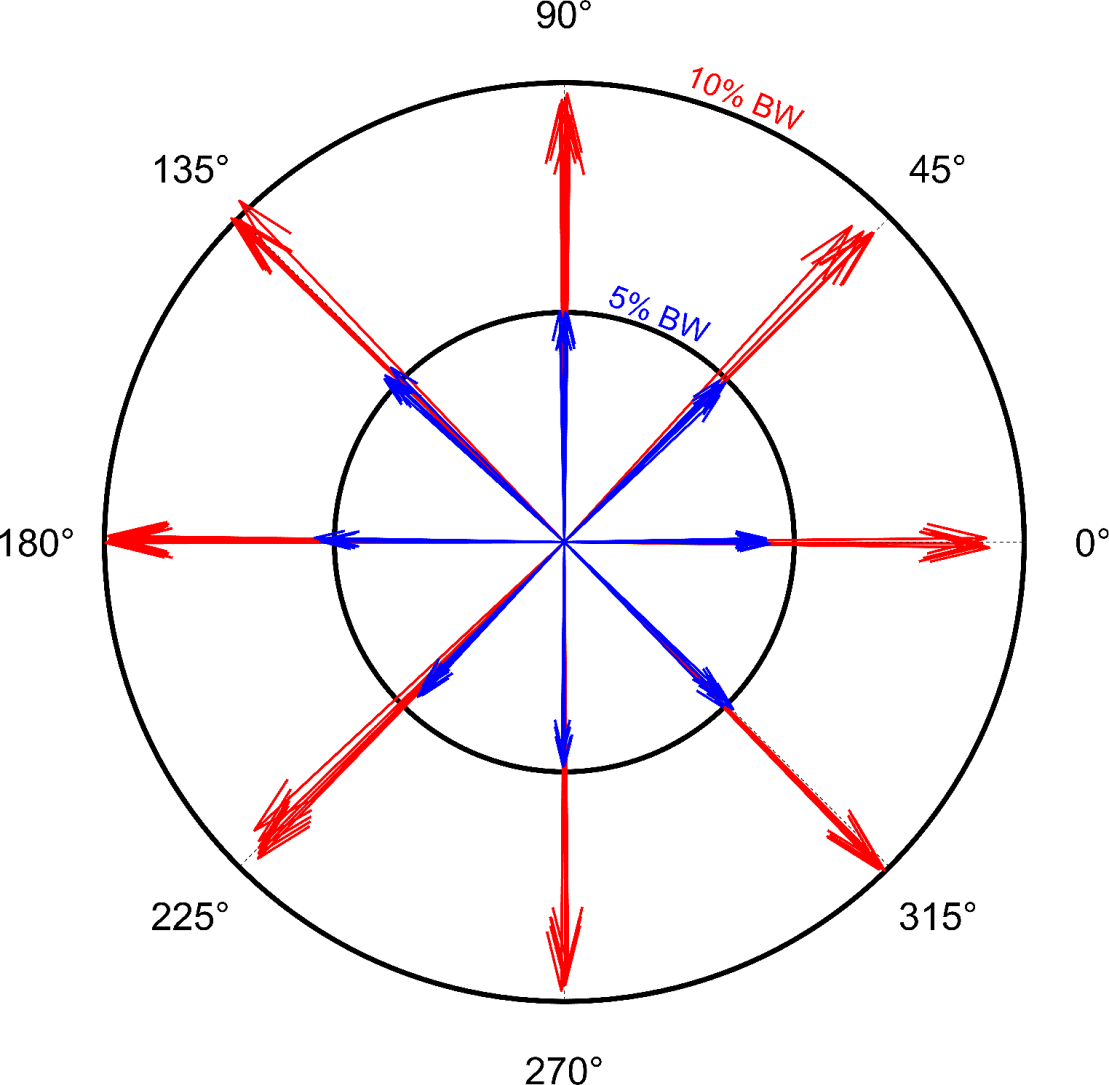
nGRF of a representative participant. Each arrow indicates the pulling force obtained from each trial for both the pulling direction load conditions. The shorter the distance from the arrow to the corresponding circle, the higher the quality of the task

The resultant forces applied to the participants in all the investigated conditions are reported in Fig. 2 for a typical subject. Force directions and magnitudes reported in Fig. 2 are consistent with those requested during the task, suggesting that the participants could properly exert the requested force in the correct direction.

**Fig. 3:**
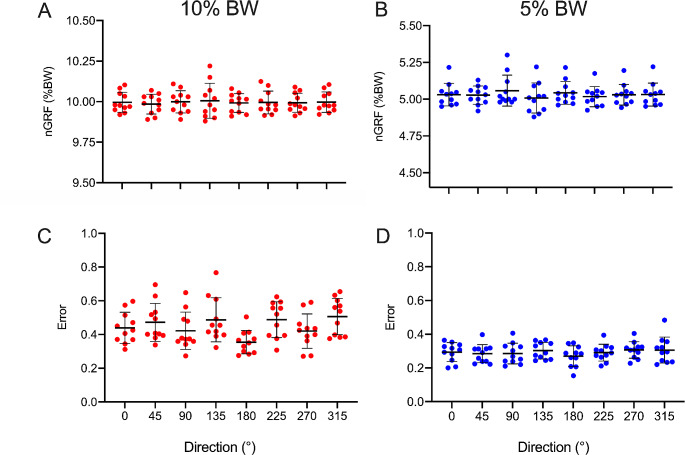
nGRF (upper panels, **A** and **B**) and Error (lower panels, **B** and **D**) reached by each participant in all the investigated pulling directions in the two load conditions (10% and 5% BW on the left and right panels respectively). Each dot refers to the mean value obtained by one participant any given direction. Mean values and standard deviation are also reported as thick and thin horizontal lines, respectively. For the upper panels indicating the nGRF: the closer the dot to the corresponding percentage (10% and 5% BW) the higher the quality of the trial. For the lower panels indicating the Error: perfect task refers to an error of 0 (zero). For the lower panels indicating the *Error*: values larger and lower than 1 indicated that the participant was outside or inside the target, respectively

Figure 3 reports the *nGRF*, normalized with body weight (% BW), and the *Error* obtained during the *steady-state phase* for all the participants and in all the investigated conditions. The two-way ANOVA found significant effects for *Load* (F_1,10_ = 8294.4, *p* < 0.001) on *nGRF*, while no significant effect of *Direction* (F_7,70_ = 1.033, *p* = 0.42) or interaction effect were observed (F_7,70_ = 1.073, *p* = 0.39). Overall, the *Error* was lower than 1 mm across conditions. The *Error* showed a main effect for *Load* (F_1,10_ = 88.72, *p* < 0.001) and *Direction* (F_7,70_ = 4.26, *p* = 0.032), while no interaction effect (load × distance) was observed (F_7,70_ = 2.68, *p* = 0.055). In this regard, the condition at 180° showed lower error when compared to 0°, 45°, 135°, 225° and 315° conditions.

### Muscle synergies

We investigated the synergistic organization of muscle patterns during the maintenance of the upright standing posture under the action of external pulling forces in the horizontal plane. The high-pass filtered, rectified and low-pass filtered of EMG waveforms recorded from 16 lower limb and trunk muscles were aligned on time and analyzed during the 10-seconds steady-state phase.

**Fig. 4:**
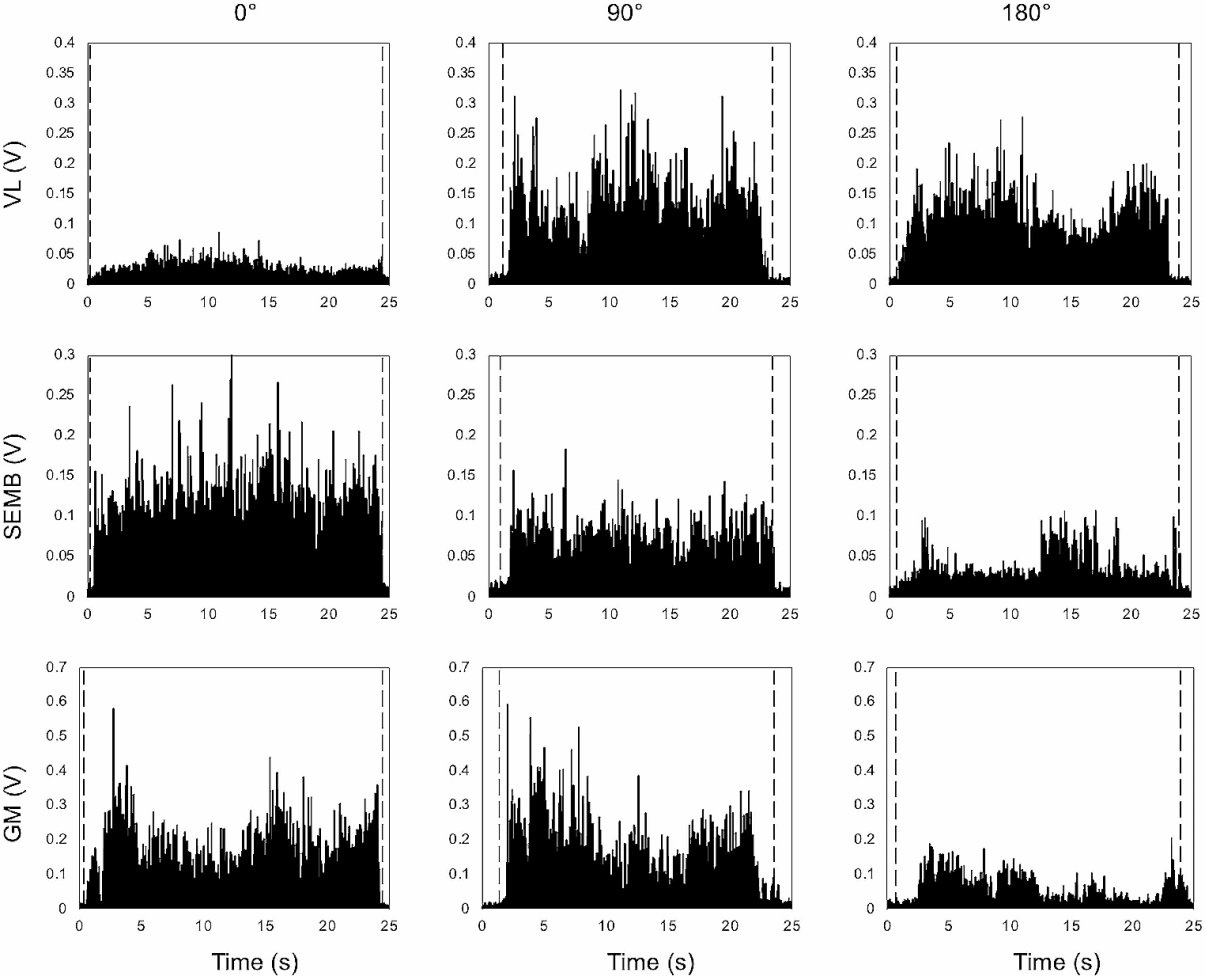
Example of raw EMG signals after rectification for a representative participant in three investigated pulling directions (0°, 90°, 180°, and 225°) for one load condition (10% of body mass). Vertical dashed lines refer to the start and end of the steady-state phase. GM: medial gastrocnemius; SEMB: semimembranosus; VL: vastus lateralis

**Fig. 5:**
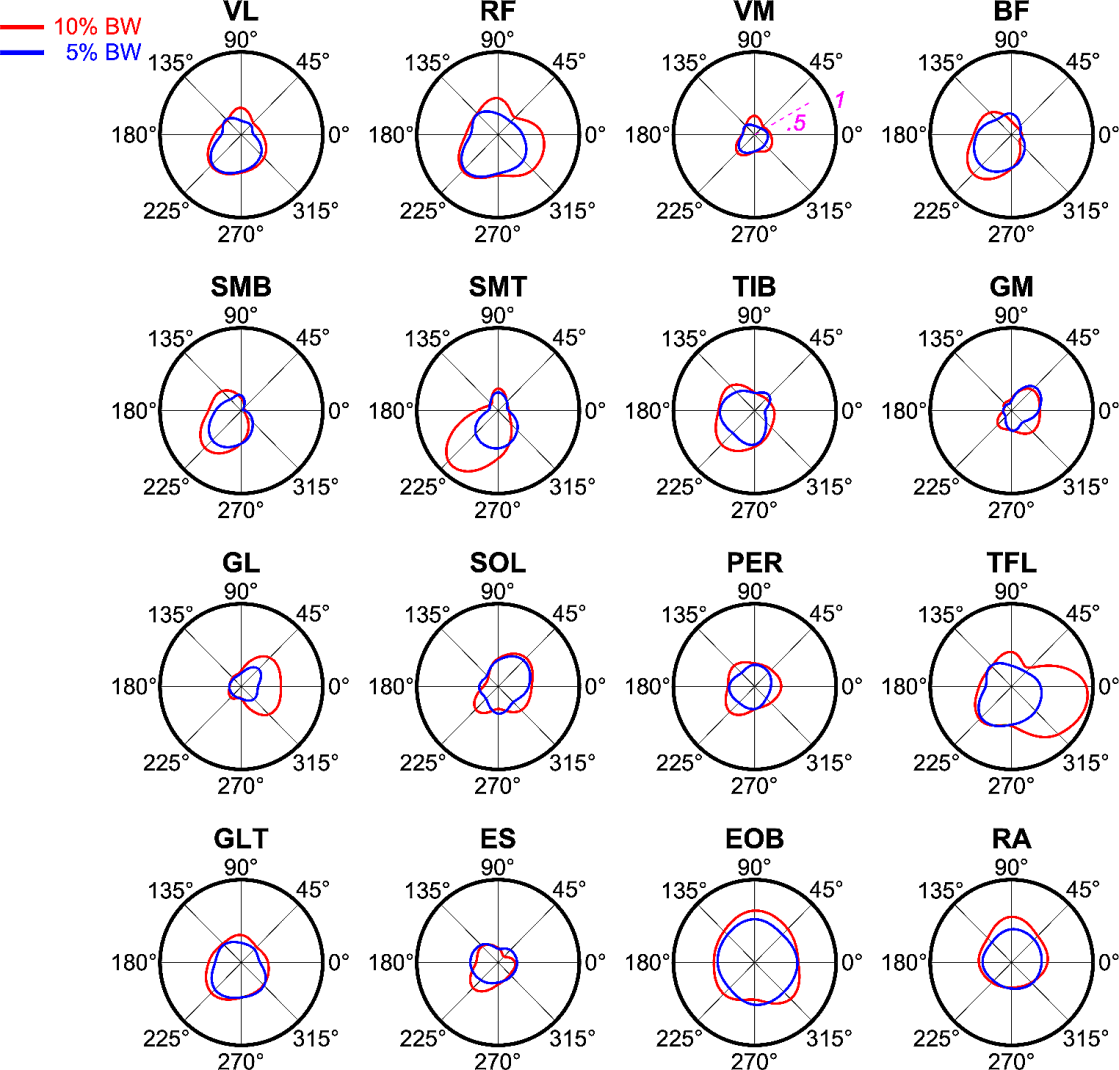
Tuning of averaged normalized EMGs in the investigated pulling directions for participant P8. Black external circle represents the angular direction. The EMG values of each muscle during the steady-state phase were normalized to the maximum value across all loads and directions connected by a periodic cubic spline interpolation curve. Note that, after this normalization, all the EMG values were constrained to the range from 0 to 1 (see the unit scale in the plot VM muscle plot)

For muscle synergies extraction, the EMG waveforms of each muscle were normalized to their maximum across all loads and directions, then the mean value throughout the steady-state phase was calculated (see Fig. 4 for an example of the raw data). Figure 5 shows the averaged (over 5 trials) normalized activation of each muscle for all pulling directions and loading conditions connected by a periodic cubic spline interpolation curve in a representative participant. The muscle activity appeared modulated by the direction of the pulling forces. The tonic activity during the upright *steady-state phase* changed with the direction of the force, and for a given direction, increased in amplitude when increasing the load. Our goal was to relate the changes in the activation of individual muscles with load direction and load to the modulation of few muscle synergies, the coordinated recruitment of muscle groups.

**Fig. 6:**
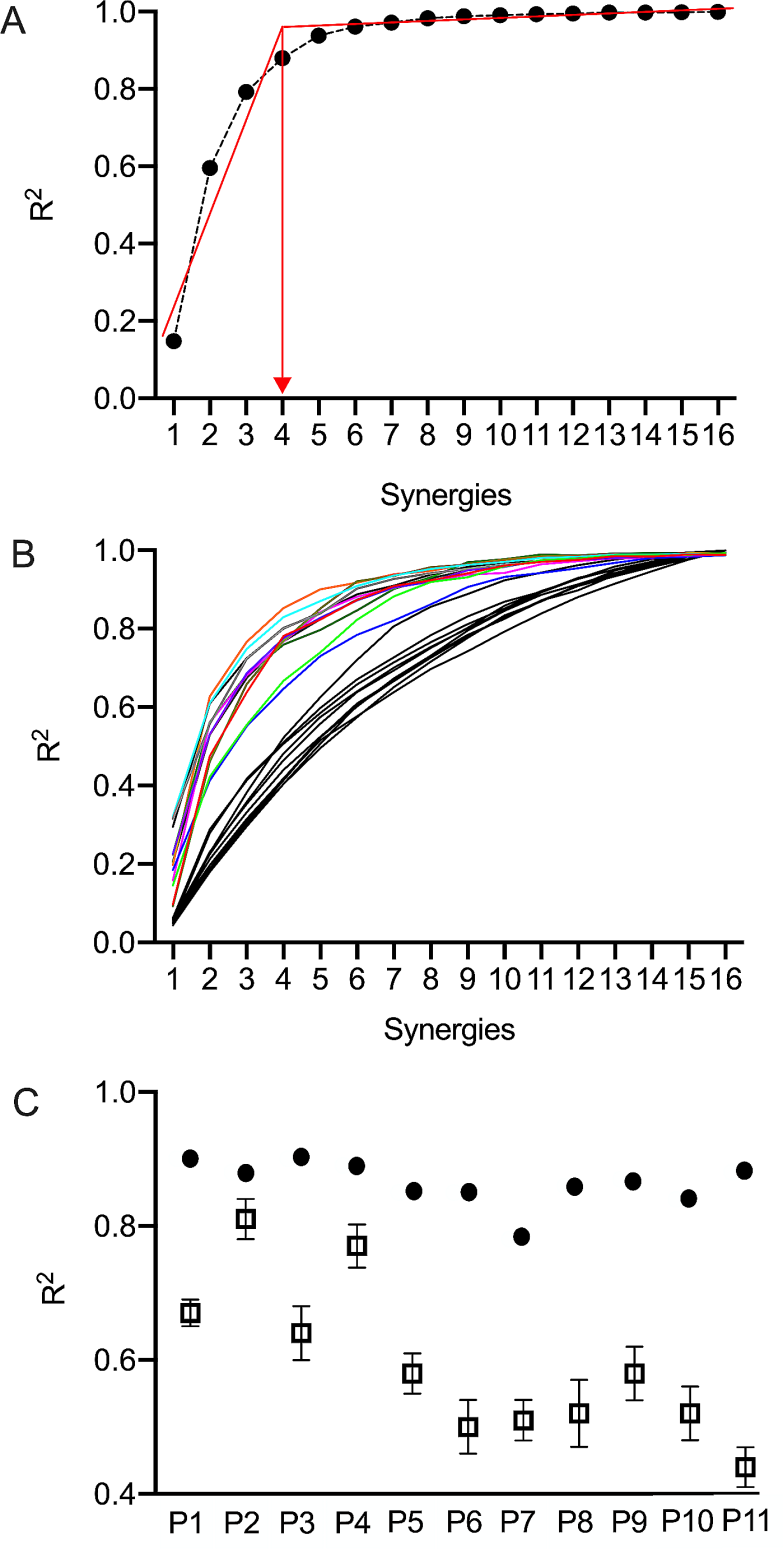
**(A)** The number of synergies were selected as the curve of R^2^ values, as a function of the number synergies extracted, showed a change in slope, indicating that adding additional synergies did not significantly improve the accuracy of the reconstruction. Here in the example, from a representative participant, 4 is the optimal compromise between model accuracy and parsimony in the choice of the number of synergies; **(B)** Colored lines: curve R^2^ values of the synergies extracted from the actual dataset (each color represents one participant), Black lines: curve R^2^ values of the synergies derived from structureless simulated data; **(C)** A much larger fraction of the total variation (R^2^) is explained by the extracted synergies from the actual dataset (black filled dots) compared to the synergies extracted from simulated structureless data (black unfilled squares, mean ± SD over 100 runs)

For each participant, we extracted sets of one to sixteen synergies using NMF and we selected the number of synergies as the number at which the curve of R^2^ showed a significant change in slope (Fig. 6-A). An average of 5.6 ± 1.3 synergies were selected in all the participants with an average reconstruction R^2^ value of 0.87 ± 0.03, indicating that the variations in the postural muscle patterns were well captured by the selected number of synergies (Fig. 6-B). The synergy extraction by normalizing the EMG values to the maximum value of 5% and 10% BW separately, yielded consistent results across all participants, with the same number of synergies selected (R^2^ value of 0.85 ± 0.04).

We assessed whether the extracted muscle synergies represented structural elements of EMG activations and were not influenced by methodological biases by comparing the reconstruction error associated with the synergies extracted from the actual dataset to that of synergies derived from structureless simulated data (see Material and Methods section) (Fig. 6-B). The paired t-Test found that, across all eleven participants’ datasets, the R^2^ value corresponding to the selected synergies from the real data consistently exceeded that of the corresponding simulated data [t(10) = 8.74, p = < 0.001] (Fig. 6-C), thereby indicating that the extraction algorithm successfully captures invariant relationships within the muscle waveforms and does not merely conform to the individual muscle waveforms.

**Fig. 7:**
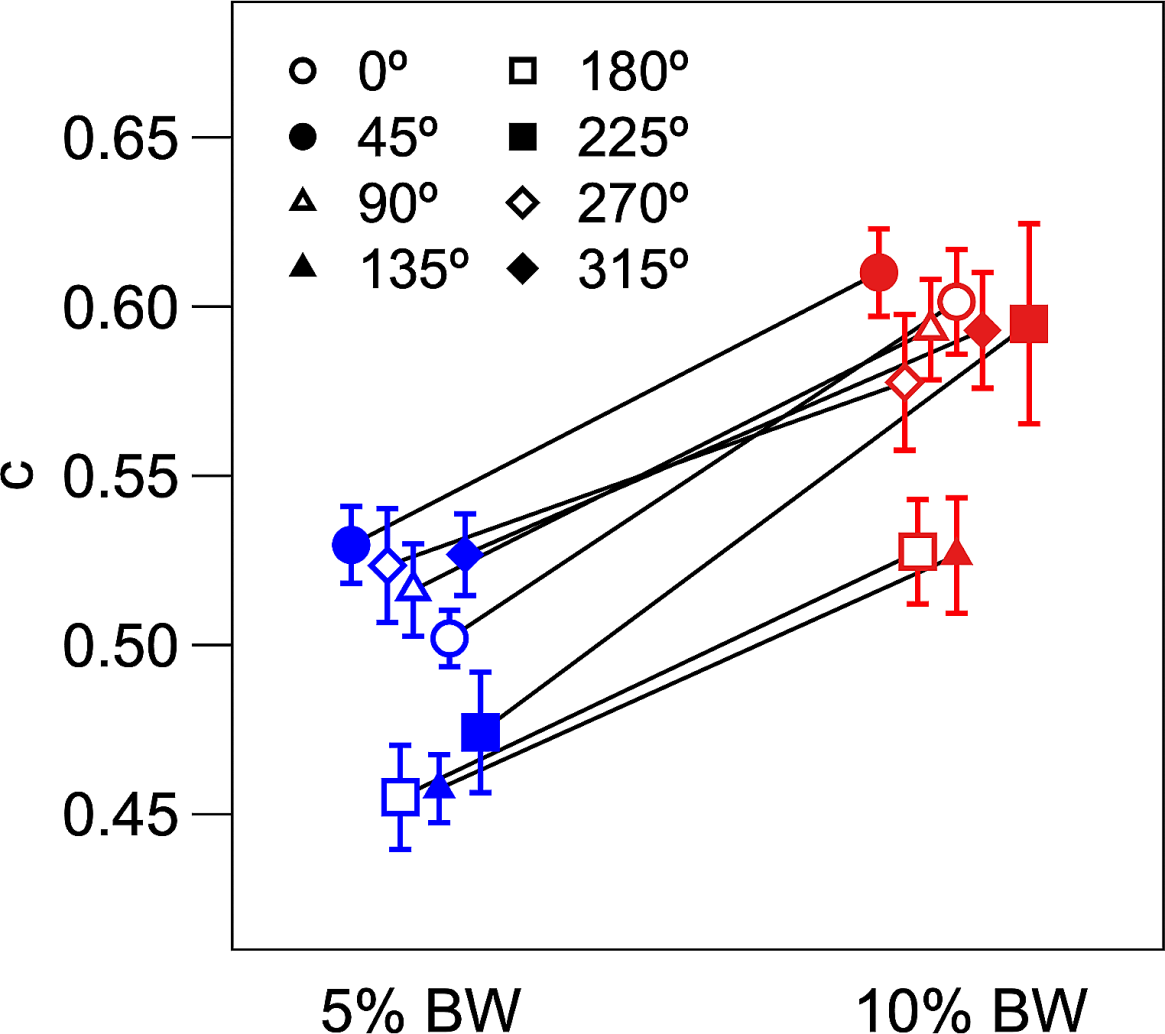
Activation coefficients of muscle synergies for the investigated pulling directions (indicated with different symbols) between load conditions (5% BW and 10% BW on the left and right, respectively). Symbols represent the mean values across participants and the bars indicate the standard error. Activation coefficients of synergies were significantly higher in the 10% BW compared to 5% BW pulling load condition for all the investigated directions

By and large the postural synergies expressed a specific balance in the activation of the muscles and were modulated in amplitude between the loading conditions and across the pulling directions. Precisely, the ANOVA found significant effects for *Load* (F_1,10_ = 151.58, *p* < 0.001) and *Direction* (F_7,70_ = 5.18, *p* < 0.001) on the activation coefficients for all pulling directions. On average, the activation coefficients were greater for the loading condition 10% BW compared to 5% BW (Fig. 7).

**Fig. 8:**
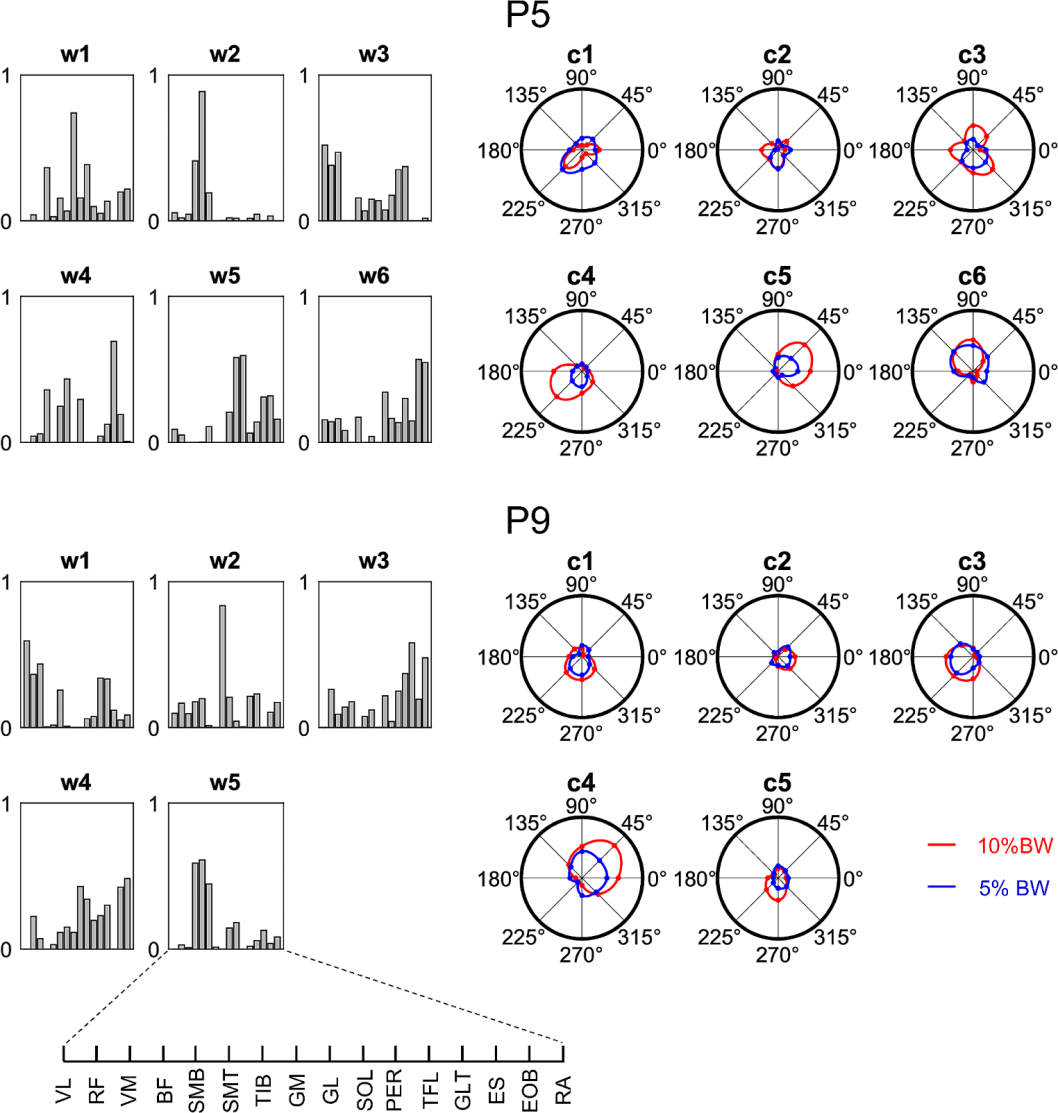
Muscle synergies (w) (left side) and activation coefficients (c) of muscle synergies (right side) for two representative participants (P5 and P 9). Activation coefficients of each muscle synergy were averaged over the 5 trials for each investigated load condition and pulling direction connected by a periodic cubic spline interpolation curve

Distinctive features of postural synergies can be observed in Fig. 8 for two representative participants (P5 and P9). Both participants show directional tuning of activation coefficients with different degrees of amplitude modulation between loading conditions (i.e., 10 and 5% BW) for most muscle synergies. It can be noticed that modulation of the maximum amplitude of the directional tuning between loading conditions is large for some synergies (P5: w4 and w5; P9: w1, w3, and w4), while small for others (e.g., w2 for both P5 and P9).

**Fig. 9:**
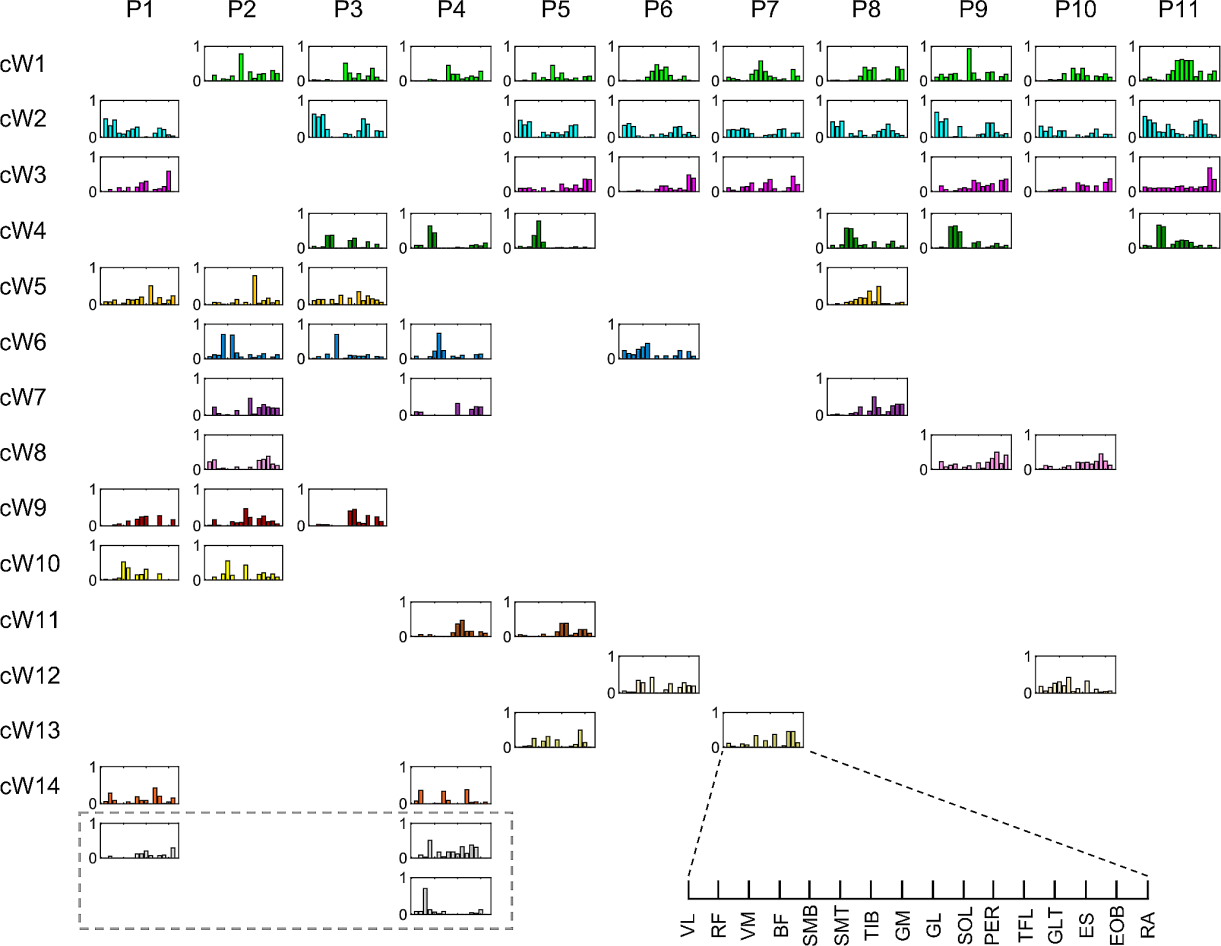
Muscle synergies in participants. Extracted muscle synergies were sorted and grouped (cWi) using hierarchical cluster analysis. Each row indicates similar muscle synergies among participants. Participant-specific muscle synergies are indicated inside the dashed line box at the bottom

To compare synergies extracted from different participants, we partitioned the set of 62 synergies extracted from all participants into 17 clusters, according to their similarity, with a clustering algorithm requiring that no more than one synergy from the same participant is included in each cluster (Fig. 9) (see Material and methods for details). A cophenetic correlation coefficient of 0.723 was obtained for the hierarchical clustering tree, indicating that the clustering solutions reasonably matched the initial observations. We also considered an alternative method for determining the number of clusters, based on the vertical differences between nodes in the dendrogram (see Material and methods for details), resulted in a total of 18 clusters. Notably, only an additional dissimilarity was observed for synergy cW10 between participants P1 and P2 (refer to Fig. 9), further supporting the validity of outcomes obtained through the employed constrained method, which limits the inclusion in each cluster of no more than one synergy from the same participant.

The first two clusters, cW1 and cW2, contained synergies from 10 to 9 out of 11 participants, respectively. Specifically, synergies in cluster cW1 had a large activation mainly in the calf muscle and hip extensors, while synergies cluster cW2 had a large activation largely in knee extensors. cW3 and cW4 clusters contained, separately, synergies from 7 to 6 participants. Synergies in cluster cW3 were characterized by a large activation in trunk and ankle plantar flexors muscles, and synergies in cluster cW4 showed a large activation primarily in knee flexors muscles. To follow, cW5 and cW6 contained synergies from 4 participants, and the remainders were contained by 2 to 3 participants or characterized by subject-specific synergies.

**Fig. 10:**
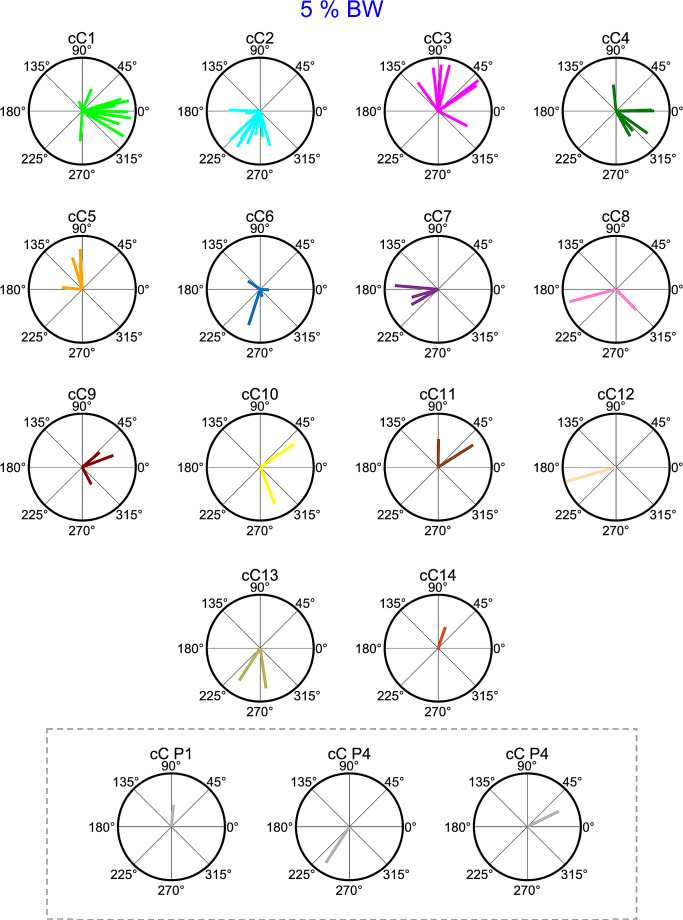
Polar plots of preferred directions (θ^PD^) of sorted activation coefficients of muscle synergies (sC) for 5% BW pulling load condition based on hierarchical cluster analysis. Participant-specific activation coefficients are indicated inside the dashed line box at the bottom. In each plot, the length of the radial segment indicates the r^2^ value of the cosine fit of the dependence of amplitude coefficients on pulling direction

**Fig. 11:**
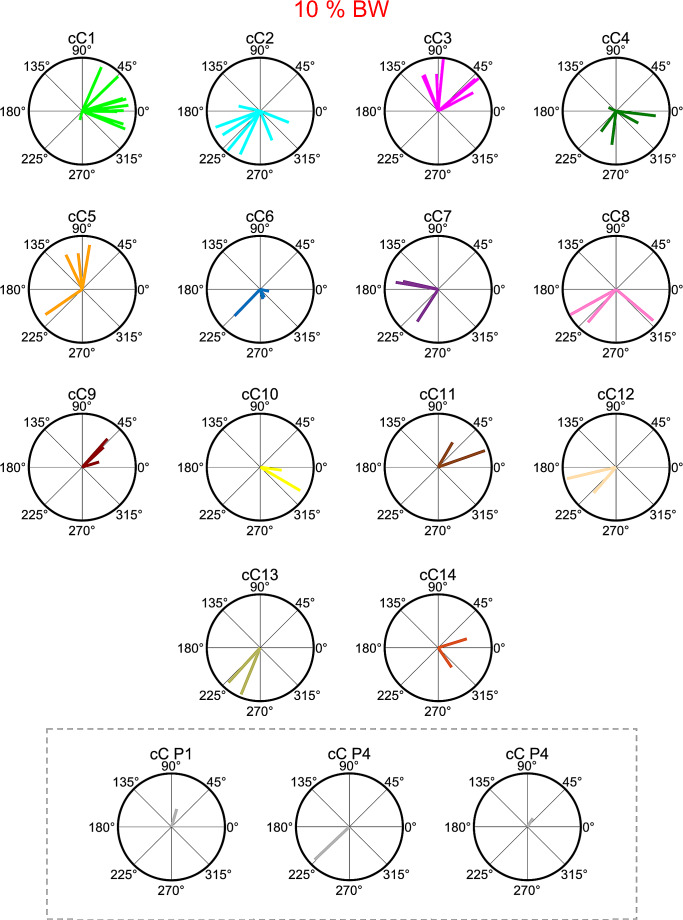
Polar plots of preferred directions (θ^PD^) of sorted activation coefficients of muscle synergies (sC) for 10% BW pulling load condition based on hierarchical cluster analysis. Participant-specific activation coefficients are indicated inside the dashed line box at the bottom. In each plot, the length of the radial segment indicates the r^2^ value of the cosine fit of the dependence of amplitude coefficients on pulling direction

The directional tuning of the recruitment amplitude of the synergies was in most cases well captured by a cosine function. To perform a statistical analysis of the dependence of the synergy amplitude coefficients on the pulling direction and loads, we fitted the data for individual trials with the synergies extracted from averaged data (see Material and methods). We performed a linear regression of the synergy amplitude coefficients for individual trials, separately for each participant and load, on the pulling directions. The regression, corresponding to the fitting of the cosine function (see Material and methos), was significant for most of the participants, loads and synergies (95/124 cases, 76.6%, *F* test, *p* < 0.05). The median distribution of the r^2^ values for all the regressions was 0.69. Figures 10 and 11 show the polar plots of the θ^PD^ of the amplitude coefficients from clustered synergies for loads conditions 5% BW and 10% BW respectively. In each plot, the length of the radial segment represents the r^2^ value of the cosine fit of the dependence of amplitude coefficients on pulling force directions and loads. The *AngDev* of the cosine tuning across pulling loads, for each participant and synergy, was relatively small (Figs. 10 and 11). Specifically, the mean of the distribution of *AngDev* values, for all participants and synergies was 32.7º (± 3.9º SEM), indicating that the directional tuning of the synergy amplitude coefficients did not change markedly with the pulling load across participants.

**Fig. 12:**
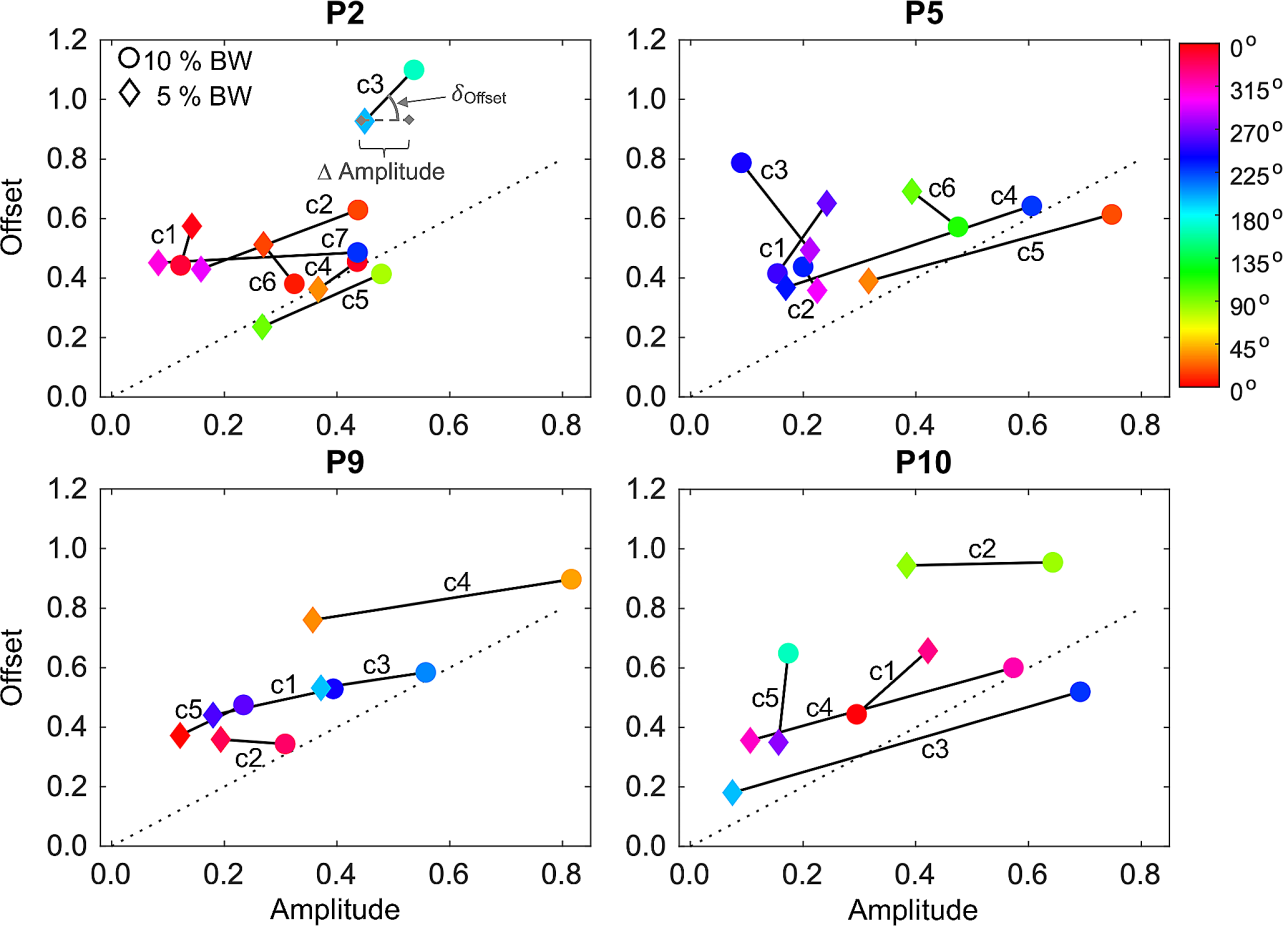
Directional tuning of synergy amplitude coefficients for representative participants, P2, P5, P9 and P10. The circles represent the 10% BW pulling condition, while the diamonds represent the 5% BW pulling condition. the color map (at the top right) specifies the θ^PD^ of the directional tuning. A graphical illustration of ∆ Amplitude and 𝛿_Offset_ computation for a single participant and synergy is shown in the plot reporting participant P2 and synergy c3

**Fig. 13:**
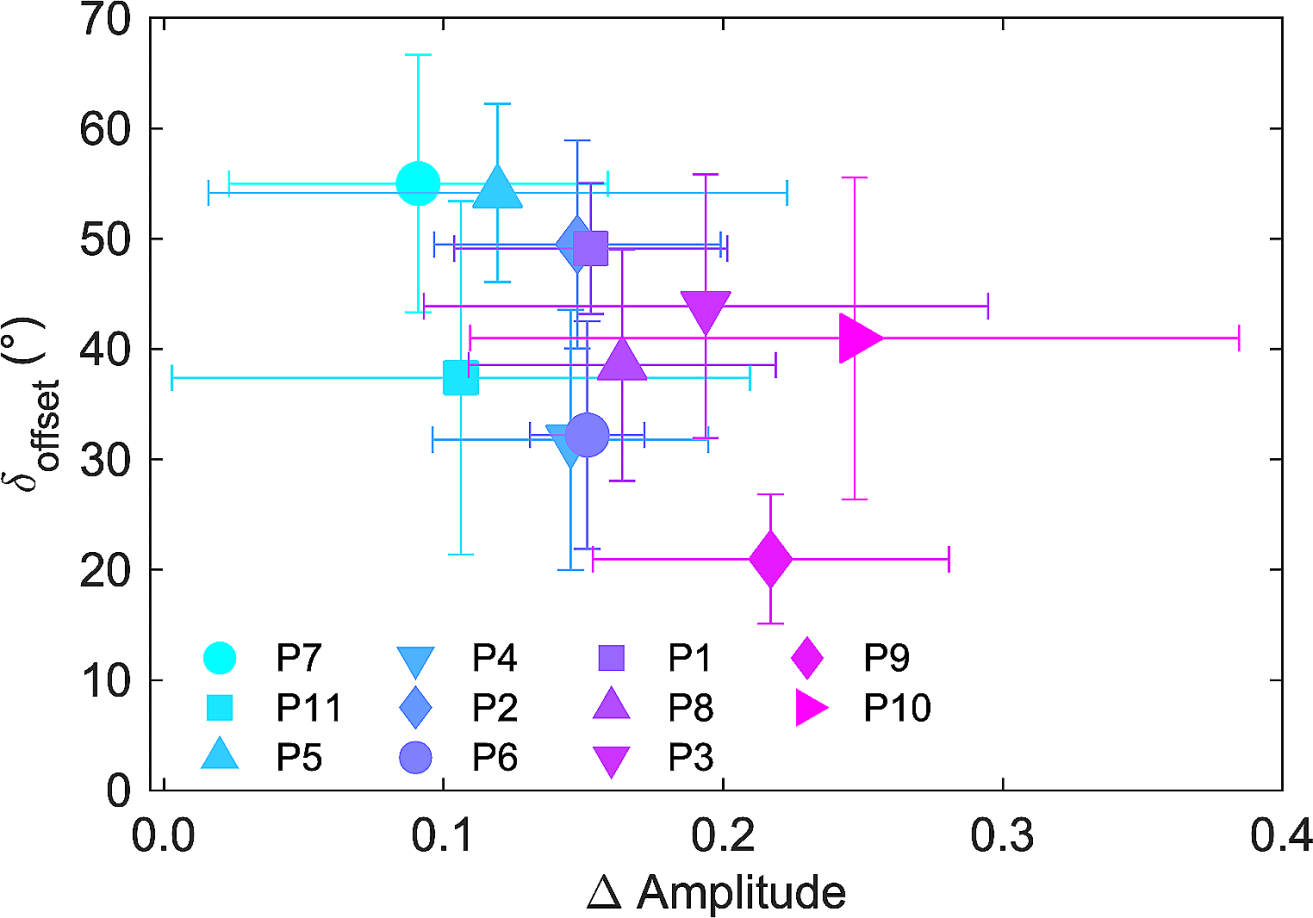
The relation between ∆ Amplitude and 𝛿_Offset_ of the directional tuning for all participants. Participants are represented by different symbols and colors, and the bars indicate the standard error

When looking at the directional tuning of synergies within individual participants, there was a noteworthy subject-specific distribution in the values of *Amplitude* and *Offset* parameters of the cosine function (see Material and methods for details), for synergies coefficients in both the pulling load conditions. Figure 12 shows the plots of *Amplitude* vs. *Offset* parameters of cosine function separately for synergies and pulling loads in four representative participants (P2, P5, P9 and P10). The color of the symbols specifies the θ^PD^ (according to the color map shown at the top right) for each synergy and pulling load. For each participant, it can be observed that there are synergies with a large modulation in the *Amplitude* of the cosine tuning function between pulling load conditions and minimal changes in the *Offset* parameter, while other synergies displayed a small modulation of the *Amplitude* with pulling loads along with a large change in the *Offset* parameter. To summarize the changes between the two pulling load conditions in the directional tuning of the synergies among all the participants, we calculated the mean difference in *Amplitude* (∆ *Amplitude*) across all the synergies for each participant, and the mean angle of inclination (𝛿_*Offset*_) of the vector formed by the difference in *amplitude* and the difference in *Offset* (see Fig. 12, participant P2 and directional tuning of c3 for details in the computation). Interestingly, the distinctive modulation between *Amplitude* and *Offset* parameters for each synergy is noticeably heterogenous among participants, as shown in Fig. 13 where participants showed a broad distribution of ∆ *Amplitude* and 𝛿_*Offset*_. For example, participant P9 shows a distinct modulation on *Amplitude* in most of the synergies (but predominantly for c1, c3, and c4) with minimal change in *Offset* parameter between pulling load conditions, resulting with a small 𝛿_*Offset*_. Conversely participant P5 shows a meaningful modulation on *Amplitude* only for 2 out of 6 synergies (c4 and c5), which resulted with a reduced ∆ *Amplitude* and larger 𝛿_*Offset*_ (see Fig. 13).

## Discussion

The objective of this study was: (1) to extract synergies of muscle activity in leg and lower-back muscles underlying the generation of multidirectional isometric force during maintenance of upright standing posture; and (2) to investigate the structure and modulation of muscle synergies involved in such whole-body isometric force generation task.

As far as the experimental set-up is concerned, the participants were able to produce reaction forces at the ground in response to the direction and amount of horizontal pulling forces enabling them to keep the verticality of standing posture and stabilize the CoP displacement (Figs. 2 and 3). This allowed us to investigate the muscle synergies underlying the generation of multidirectional isometric force during upright standing posture.

Our findings revealed that the combinations of a limited set of muscle synergies effectively captured the orchestration of muscle activation patterns, as evident in the comprehensive utilization of the entire body to counteract forces applied to the trunk while concurrently sustaining equilibrium in a static standing posture. Additionally, the scaling of the recruitment amplitude of these synergies exhibited a notable correlation with both the direction and magnitude of the applied pulling forces. Remarkably, an unexpected discovery surfaced: a participant-specific variance in the cosine directional tuning parameters of the synergy amplitude coefficients. This observation implies the presence of distinct individualized neuromechanical strategies employed to stabilize the whole-body posture.

### Muscle synergies contribution in quiet standing posture

Previous findings have suggested that the CNS simplifies motor commands by activating muscles through flexible combinations of fixed synergies, where each synergy is defined as a set of muscles recruited by a single neural command signal (d’Avella and Bizzi 2005; Bizzi et al. 2008; Bizzi and Cheung 2013; Ting et al. 2015). Accordingly, muscle synergies represent a neural control strategy to overcome the complexity of the control of a redundant musculoskeletal system by combining basic control of modules which provide stable and predictable motor outputs (d’Avella et al. 2003; Bizzi et al. 2008; Ting et al. 2015). Experimental evidence has shown that individuals exhibit consistent motor synergies in seemingly variable muscle activation patterns across multiple muscles during externally induced or self-triggered postural responses (Krishnamoorthy et al. 2003; Torres-Oviedo and Ting 2007, 2010; Klous et al. 2010; Piscitelli et al. 2017), as well as with voluntary migration of the CoP while standing (Kubo et al. 2017; Hagio et al. 2022).

Our data showed that an average of 5 or 6 muscle synergies were sufficient to account for all muscles EMG waveforms associated with changes in the amount and direction of pulling forces (Fig. 5). Comparable numbers of synergies have found to account for the data variability in similar upright standing postural tasks (Kubo et al. 2017; Hagio et al. 2022) as well as during postural responses (Torres-Oviedo et al. 2006; Torres-Oviedo and Ting 2007, 2010; Safavynia and Ting 2013). We have shown that the recruitment of a small number of synergies scaled with pulling load and explained to a large extent the spatial characteristics of the muscle patterns underlying the generation of multidirectional isometric force during upright standing. Each synergy comprises the coordinated activation of specific muscles group, basically including proportional bursts (i.e. weights) of some muscles with specific biomechanical functions in response to the pulling actions of the external load, see for example the dorsal muscles of lower limbs of c4 for P9 in (Fig. 8). Most of the synergies identified in the participants were composed of both lower limb and thigh or trunk muscles related to the stabilization of ankle, knee, and hip joints. Thus, our results are in accordance with previous findings suggesting that the coexistence of dynamic interaction of the lower limb and hip joints is instrumental to generate precise and flexible control of human standing posture (Kuo 1995; Krishnamoorthy et al. 2003; Creath et al. 2005; Alexandrov et al. 2005; Pinter et al. 2008; Günther et al. 2009; Li et al. 2012).

Each muscle synergy was activated for specific pulling directions, as demonstrated by the directional tuning of the cosine functions (Figs. 10 and 11), which signifies that muscle synergies were not merely grouped by anatomical classification but mostly by function. This finding indicates that muscle synergies not only reduce the complexity of the control of a redundant musculoskeletal system but also generate specific force vectors, necessary to control the CoP fluctuations, that cannot be generated by a single muscle (Borzelli et al. 2013; Berger et al. 2013; Gentner et al. 2013; Kubo et al. 2017; Borish et al. 2021).

Though support for the encoding of muscle synergies by the CNS is mainly indirect, some evidence at the neurophysiological level exists. Although muscle synergies in upper and lower limbs may have different neural substrates, common principles likely underlie their recruitment and modular organization. Studies stimulating the cortex and spinal cord have reported correlated neural outputs across motor pools (Saltiel et al. 2001; Overduin et al. 2012, 2015). It has been suggested that muscle synergies in reaching and hand movements are organized at the level of corticospinal neurons (d’Avella et al. 2011; Gentner et al. 2013; Berger et al. 2017; Tardelli et al. 2022) and premotor interneurons in the spinal cord (Takei and Seki 2013; Takei et al. 2017), whereas muscle synergies in lower limbs have been proposed to be organized at the spinal level (Hart and Giszter 2004, 2010; Cheung et al. 2005; Kargo et al. 2010). However, a recent study focused on investigating cortical representations of muscle synergy involved in single-leg balance control and examined changes in cortico-synergy coherence accompanying short-term balance training (Zandvoort et al. 2019). The results demonstrated the presence of muscle synergies in the motor cortex, particularly in the paracentral lobule, known for the representation of lower extremities, which suggests that the neural organization of muscles synergies for standing postural control is shared between the cortex and the spinal cord.

### Muscle synergies similarity across participants

The hierarchical cluster analysis indicated that few muscle synergies were common among participants. The first cluster cW1 contained synergies from 10 out of 11 participants, with a clustered directional tuning (cC1) in the right anterior direction. The second cluster cW2, which contained synergies from 9 out of 11 participants, had a directional tuning (cC2) in the left posterior direction, while cW3 contained synergies only from 7 out of 11 participants with the directional tuning (cC3) mainly in the anterior direction (Figs. 10 and 11). The following six clusters contained synergies from between six (cW4) and three (cW7-cW9) participants. The remaining clusters contained synergies from a maximum of two participants. Even though a different computational method was employed to quantify the similarity of synergies, a comparable dissimilarity in muscle synergies was identified in a prior study where participants were instructed to shift the CoP in different directions by bending their body around the ankle joint (Kubo et al. 2017). Thus, our results, along with previous findings, indicate that individuals use a subjective combination of muscles to produce a multidirectional force vector during balance maintenance in the upright body position.

While muscle coordination patterns are constrained by the biomechanics of the musculoskeletal system (Kutch et al. 2008), individual differences in the specific patterns of muscle activity in standing balance control can vary considerably (Torres-Oviedo and Ting 2007; Torres-Oviedo et al. 2006; Bunderson et al. 2008). Individual differences of postural synergies and their invariant patterns of activity (i.e. weights), whether attributed to habitual or innate factors, can also be influenced by training experience, affecting the range of behaviors used to modify the specific structure of the synergies in an individual (Ting and McKay 2007; Torres-Oviedo and Ting 2010; Haith and Krakauer 2018). Previous research has suggested that the arrangement of the motor cortex reflects the prevailing muscle coordination patterns observed in an individual’s behavioral repertoire (Graziano and Aflalo 2007), indicating the potential for the shaping of muscle synergies that is specific to the individual and influenced by their experiences. Furthermore, (Hagio and Kouzaki 2015) noted that the variability in the action direction of the muscle synergies occurred because the direction of force produced by the muscle synergies reflects a pulling direction of recruited motor units. Indeed, if muscles synergies are organized at the level of spinal interneurons (Hart and Giszter 2010; Takei and Seki 2013; Overduin et al. 2014, 2015), it is likely that the mechanical feature of muscle synergies can vary depending on which subset of motor units are recruited since they have a broad range of pulling directions (Thomas et al. 1990; Borzelli et al. 2020). Therefore, it is suggested that individuals adjust their own muscle synergies within a certain range to find the best configuration for reducing redundancy in postural control (Wojtara et al. 2014).

### Characterization of muscle synergies in upright postural control

The modulation of the synergy directional tuning with load direction and magnitude did not occur in the same way for all synergies for each participant. Specifically, as shown in Fig. 12 for four representative participants, a subset of muscle synergies showed directional tuning that was modulated by either the direction or the magnitude of the load, while others showed a negligible directional modulation of the activation coefficients, and merely a change in the offset of the cosine function. However, there was a mixed distribution of tuned and untuned muscle synergies across the participants with a seemingly inverse relation between the average amplitude and the offset parameter of the cosine function (Fig. 13). The underlying neurophysiological function of these multidirectional equally balanced (i.e., untuned) muscle synergies is not well understood. Nonetheless, in a previous study individuals were asked whether they could voluntarily modulate muscle co-contraction levels when disturbances of different magnitude were applied (Borzelli et al. 2018). The results showed that when a higher level of co-contraction was required, the cosine function’s offset increased largely and its amplitude slightly. Regarding the muscle patterns, higher co-contraction was related to an increase in magnitude of the null space projections. The authors argued that the CNS might rely on a small number of specific muscle synergies also to generate appropriate co-contraction patterns. These specific synergies generate null space vectors which do not produce any force, or by specific combination of force-generating synergies with zero resultant force.

Notwithstanding, as far as it is concerned the role of muscle co-contractions during upright standing few aspects must be considered. Traditionally, the agonist and antagonist co-activation enhance postural stability by increasing the apparent stiffness of the involved joints (Nielsen and Kagamihara 1992; Lee et al. 2006). However, this view has been recently criticized in postural control since an increase in the joint stiffness contributes to stability of a kinematic chain only if one of the ends of the chain is fixed in space (Latash 2018). Indeed, this is unrealistic during vertical standing posture because the feet are not typically anchored to the ground. A few recent studies explored the effects of voluntary muscle co-contraction on indices of postural stability and found signs of impaired stability when the co-contraction level was persistently increased (Yamagata et al. 2019, 2021). Thus, an alternative interpretation of increased muscle co-contraction related to posture stabilization results by a co-variation of the muscle reciprocal changes in the activity of agonist–antagonist pairs and co-activation level within the referent body configuration framework depending on the biomechanical constraints induced by the task (Lee et al. 2006; Ambike et al. 2016; Bertucco et al. 2021; Cesari et al. 2022). Specifically, within the idea of movement control with changes in referent body configurations (RC hypothesis) muscle reciprocal activation of agonist–antagonist corresponds to the coordinate where the resultant moment of force in the joint is zero, while muscle co-activation reflects the spatial range where agonist and antagonist muscles are both activated. Changes in the muscle reciprocal activation define a coordinate in space where the joint comes to an equilibrium in the absence of external resistance, and the co-contraction level defines the range about joint coordinate with effects in the joint apparent stiffness (Latash and Zatsiorsky 1993). The idea of control with RC hypothesis have been further tested in whole-body postural control (Slijper and Latash 2000; Piscitelli et al. 2017; Bertucco et al. 2021; Cesari et al. 2022). Accordingly, it has recently been proposed that posture-stabilizing strategies could emerge from a co-existence of muscle reciprocal activations, which indicate the spontaneous migration of the CoP leading to the rambling component of postural sway, and muscle co-contraction levels that adjust as needed to ensure postural stability resulting, as whole, in strong co-variation of referent body configurations and apparent stiffness (Nardon et al. 2022). In this regards, our results of varied levels of directional tuning amplitudes from distinctive muscle synergies could reflect the coexistence of diversified neuromechanical strategies to ensure stable postures in the multi-joint system, through the control of co-contraction levels, while controlling the multidirectional fluctuations of the CoP by muscle reciprocal activations to counteract forces applied to the body in order to maintain balance in quiet standing posture (Imagawa et al. 2013; Kubo et al. 2017; Piscitelli et al. 2017). Admittedly, this is a speculative interpretation of the neuromechanical role of these differentiated muscle synergies; further investigations is needed to better clarify whether these synergies explain the underlying voluntary modulation of co-contraction of muscle patterns during the generation of isometric forces in quiet upright standing (Borzelli et al. 2018).

### Limitations

EMG activity from lower limb and trunk muscles of each participant were recorded unilaterally to extract the muscle synergies. We assumed that the muscle patterns from the lower limbs and trunk of both body sides were symmetrical and mirrorlike given the geometrical arrangement of investigated pulling force directions. Muscle synergies during postural responses have been accurately identified with external perturbations applied in similar evenly spaced directions in the horizontal plane by recording the muscle activity in only one side of the body (Torres-Oviedo and Ting 2007, 2010; Chvatal and Ting 2013; Safavynia and Ting 2013). However, it might be possible that, because of the nature of experimental postural task in our study, the recording of muscle activity from only one side of the body could have not well accounted for the total data variability in the synergies’ extraction, resulting in a suboptimal spatial reconstruction of the directional tuning of synergy coefficients.

Moreover, in the present study, our objective was to delineate the muscle strategies employed with a focus on the average muscle activation necessary to uphold the steady-state postural position. Nonetheless, we acknowledge the necessity for further investigations to ascertain the specific contribution of time-varying changes in the recruitment amplitude of muscle synergies to the continuous control of CoP fluctuations during the maintenance of an upright standing posture.

## Conclusions

In this study we aimed to investigate systematically muscle synergies under multidirectional isometric force generation during maintenance of upright standing posture. Our findings showed that a small set of synergies can be used to stabilize the vertical posture by the independent modulation a small set of muscle synergies to control the inherent fluctuation of the CoP. The muscle synergies were evenly distributed on the horizontal plane, and the reduction of redundancy of the musculoskeletal system resulted in individual strategies.

Our study provides a prospective basis for potential application of voluntary and multi-directional force generation to assess muscle coordination in the context of CoP sway control during steady-state upright standing. Specifically, the examination of muscle synergies emerges as a valuable metric for motor assessment, given that alterations in the number, structure, and recruitment of muscle synergies have the potential to discern the control and coordination of postural muscles during stable upright standing among a variety of physiological and pathological changes in the nervous system (Safavynia et al. 2011; Ting et al. 2015), and link to scientific knowledge about the functions of the neural mechanisms of posture affected by injury, impairment (Milosevic et al. 2017; de Kam et al. 2018; Yang et al. 2019) or as a consequence of exercise and training (Allen et al. 2017; Ai et al. 2023). Furthermore, it lays the foundation for exploring synergy control-based approaches in human-computer interaction environments to promote recovery and improvement of motor skills in individuals with postural deficits (Berger et al. 2013, 2022; Berger and d’Avella 2014; Borish et al. 2021).

### Electronic supplementary material

Below is the link to the electronic supplementary material.

Supplementary Material 1

Supplementary Material 2

Supplementary Material 3

## Data Availability

The datasets generated and/or analyzed during the current study are available from the corresponding author on reasonable request.
