# Supplementary figures and images for: Muscle synergies for multidirectional isometric force generation during maintenance of upright standing posture
Source: Exp Brain Res. 2024 Jun 14;242(8):1881–902. doi: 10.1007/s00221-024-06866-z (PMC11252224; doi:10.1007/s00221-024-06866-z)

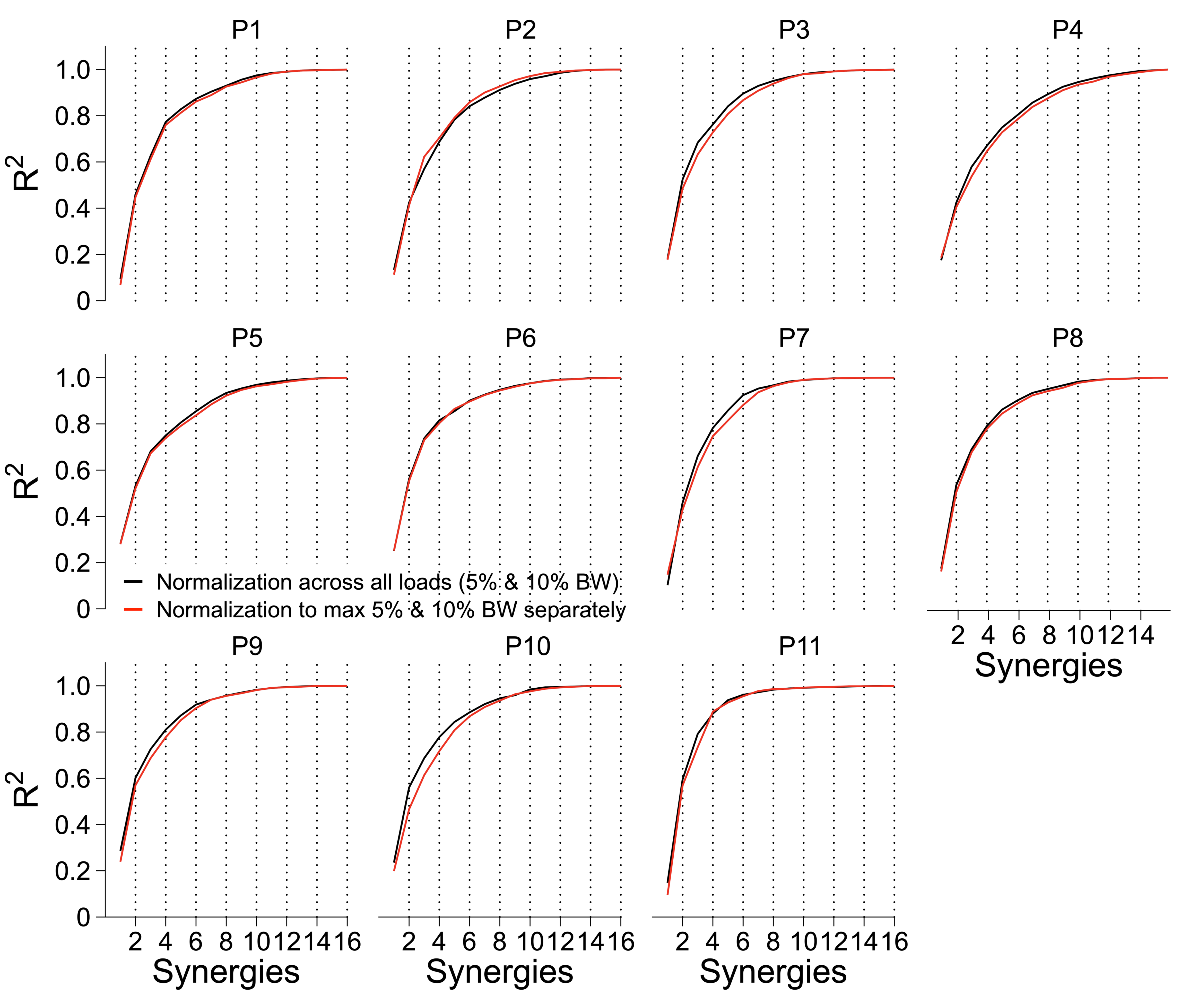

Supplement: Supplementary file 1 — Supplementary Material 1 [file 221_2024_6866_MOESM1_ESM.tif]

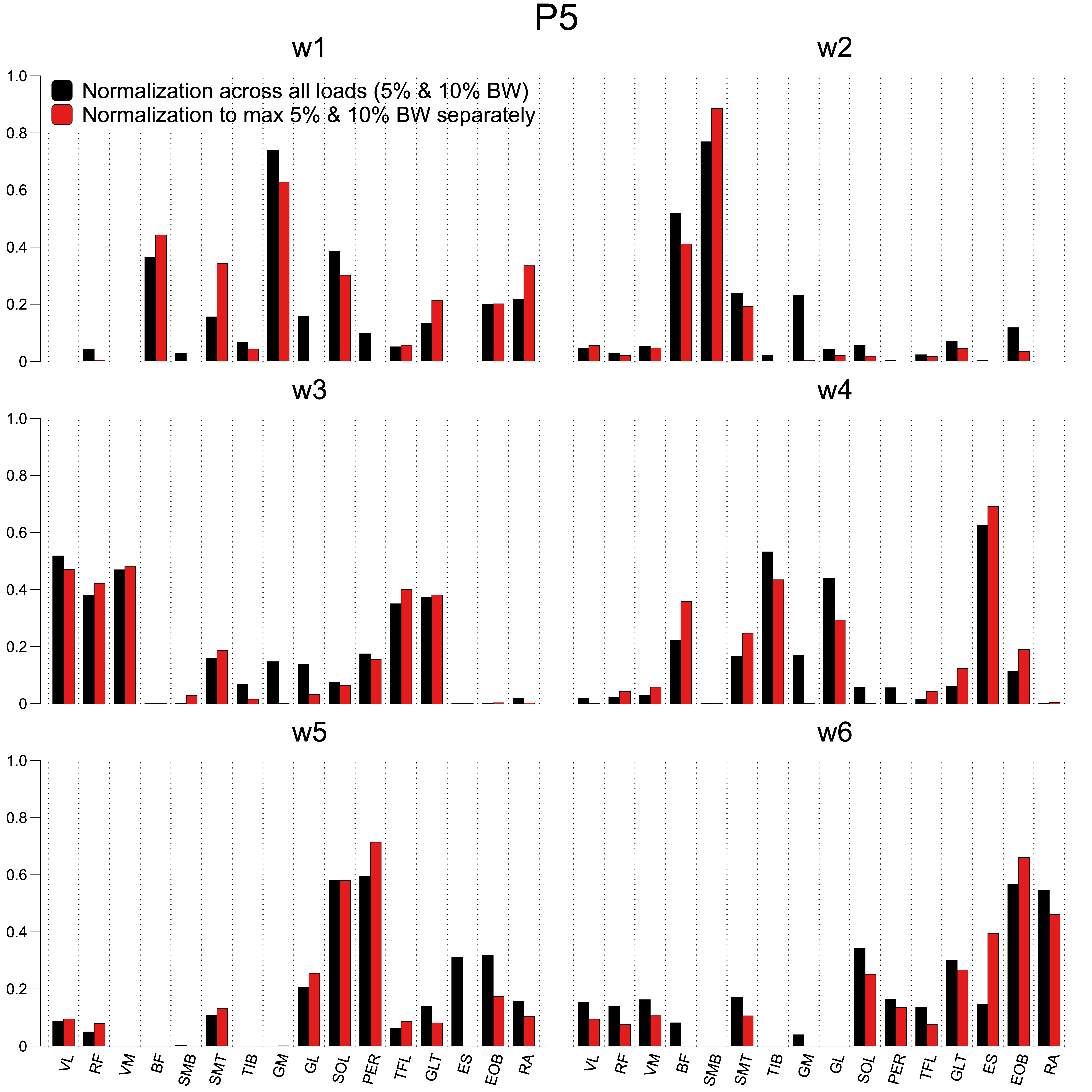

Supplement: Supplementary file 2 — Supplementary Material 2 [file 221_2024_6866_MOESM2_ESM.tiff]

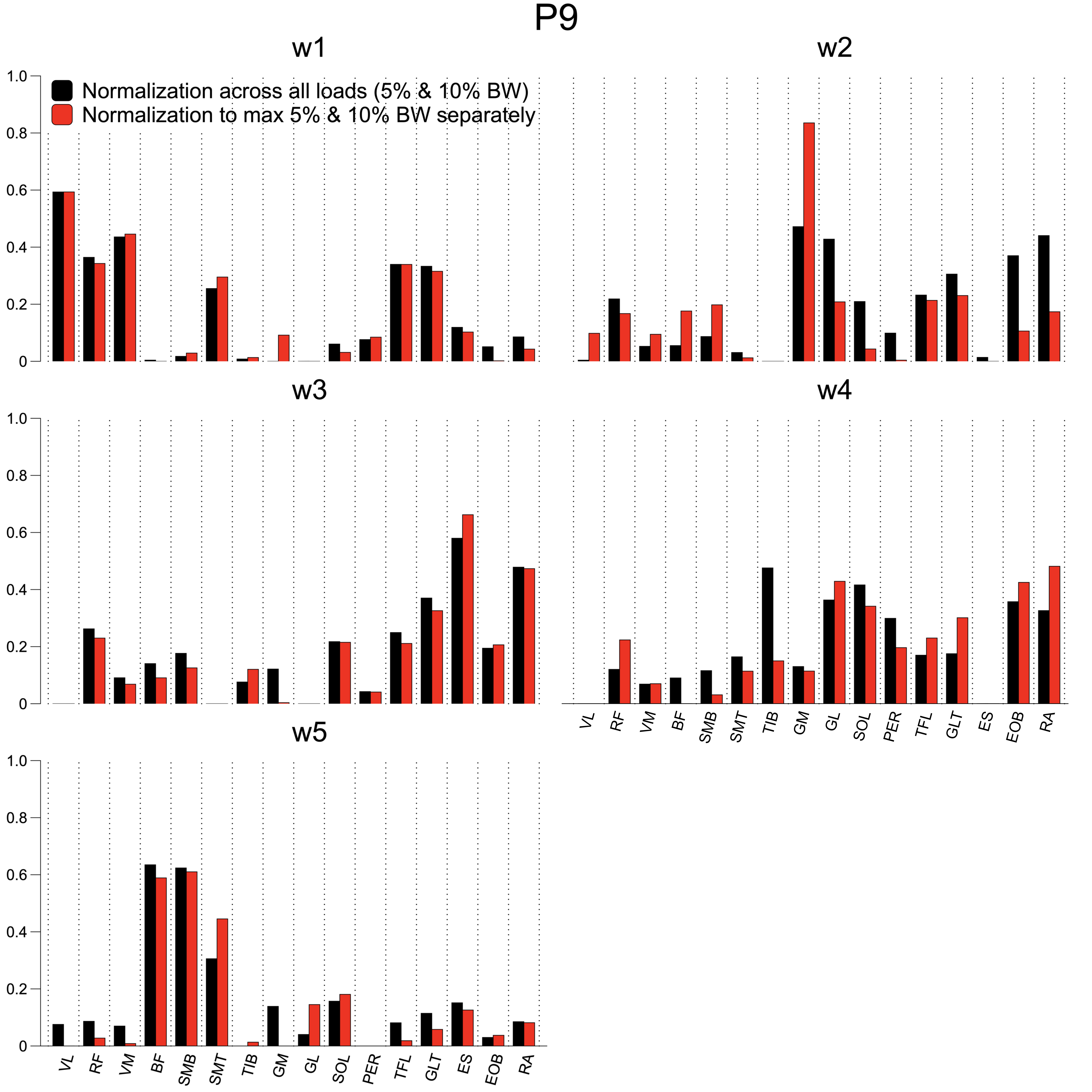

Supplement: Supplementary file 3 — Supplementary Material 3 [file 221_2024_6866_MOESM3_ESM.tif]
